# Arthroscopic removal of loose bodies using the accessory portals in the difficult locations of the knee: a case series and technical note

**DOI:** 10.1186/s13018-018-0966-z

**Published:** 2018-10-19

**Authors:** Biao Chen, Liaobin Chen, Haitao Chen, Xu Yang, Kai Tie, Hua Wang

**Affiliations:** Department of Orthopedic Surgery, Zhongnan Hospital of Wuhan University, Wuhan, 430071 China

**Keywords:** Loose bodies, Arthroscopic removal, Popliteus hiatus, Posterior compartment, Extreme lateral approach, Double-posteromedial portal

## Abstract

**Background:**

It is often technically demanding to find and remove loose bodies in several difficult locations like the popliteus hiatus and posterior compartment arthroscopically. We aim to present the technical aspects of establishing some special accessory portals to achieve arthroscopic removal of the loose bodies in these locations.

**Methods:**

From September 2010 to July 2017, 76 patients underwent removal of loose bodies in the popliteus hiatus and posterior compartment arthroscopically using some special accessory portal techniques. An auxiliary extreme lateral approach was established to remove loose bodies in the popliteus hiatus; a double-posteromedial portal was applied to handle loose body removal in the posteromedial compartment, and the posterior trans-septal portal was needed for loose body removal in the posterolateral compartment. Functional outcomes were evaluated using Lysholm score, Tegner score, and International Knee Documentation Committee (IKDC) score, respectively.

**Results:**

Seventy-six patients (24 males and 52 females, average age 54.9 ± 11.4) finished the follow-up visit at 3 weeks after surgery. There was no statistically significant difference among the three groups in demographics. All the patients were performed following the special technique. According to a comparison of knee joint scores before and after surgery, all the patients obtained good prognosis using some special accessory portals in loose body removal.

**Conclusions:**

With the help of the above accessory portals under endoscopic visualization, loose bodies in the popliteus hiatus and posterior compartment of the knee can be safely and effectively removed.

## Background

Loose bodies, also known as joint mice [1], are mostly associated with the osteochondral lesion, osteochondritis dissecans, joint degeneration, and synovial chondromatosis [2]. Clinically, pain and intermittent locking, with or without swelling and effusion of the knee, are often found in patients with loose bodies, which bring great psychological pressure to them. Furthermore, loose bodies moving freely in the joint are more likely to cause the disorder of intra-articular structure, result in severe damage to the articular cartilage, and thus aggravate the development of early osteoarthritis (OA) [3].

Arthroscopy is considered to be the standard technique for loose body removal in the knee [4–6]. Despite remarkable advances in arthroscopic technology over the past decades, loose bodies in several sections of the knee joint remain difficult to remove. Regarding popliteus hiatus, it represents an oblique anterolaterally directed tunnel, bordered by the superior and inferior fascicles of the lateral meniscus [7–9]. Popliteus hiatus appears as a potential space, pushed closed by the adjacent lateral capsule and ligaments [7]. Due to the specificity of the anatomical structure, it might be very hard not only to clearly expose the structures of the popliteus hiatus through conventional arthroscopic technique [10] but also to remove the loose bodies located in it. As for the posterior compartment of the knee joint, the posterior aspect of the proximal tibia and blind spots in knee arthroscopy are difficult to access and manipulate [5]. Furthermore, the popliteal artery lies in close proximity to the posterior compartment of the knee, and the risk of its damage remains high [11]. Besides, dynamic procedure and structural variation of neurovascular anatomy may increase the risk of an iatrogenic injury [12]. Therefore, it is still necessary to further discuss the technique of removing loose bodies in the above sections.

So far, only a few studies reported the removal of loose bodies in the posterior compartment of the knee under arthroscopy. In a technical note, Ahn et al. [3] removed the loose bodies located in the posterior compartment of the knee with the help of trans-septal portal. Pengatteeri et al. [13] in another study reported that the arthroscopic posterior–posterior triangulation technique might be useful for the management of synovial chondromatosis of the posterior cruciate ligament. However, previous techniques were inevitable to damage the posterior septum, which contained several neurovascular structures and mechanoreceptors. In addition, few literatures, up to now, specifically reported loose body removal in the popliteus hiatus. This study aims to introduce some accessory portals in the removal of loose bodies in the popliteus hiatus and the posterior compartment of the knee.

## Methods

### Patients

We retrospectively studied 192 knees that underwent arthroscopic loose body removal in the popliteal hiatus and posterior compartment from September 2010 to July 2017. After a careful review, 76 knees met our inclusion criteria. Loose bodies in 20 knees were found in the popliteus hiatus, and those in 40 knees and 16 knees were detected in the posteromedial and posterolateral compartment, respectively. Inclusion criteria were as follows: (1) loose bodies located in the popliteus hiatus or posterior compartment of the knee and (2) patients who were followed up 3 weeks after surgery. (3) The operations were performed by the same surgeon group.

### Surgical technique

#### Preoperative evaluation

**Fig. 1:**
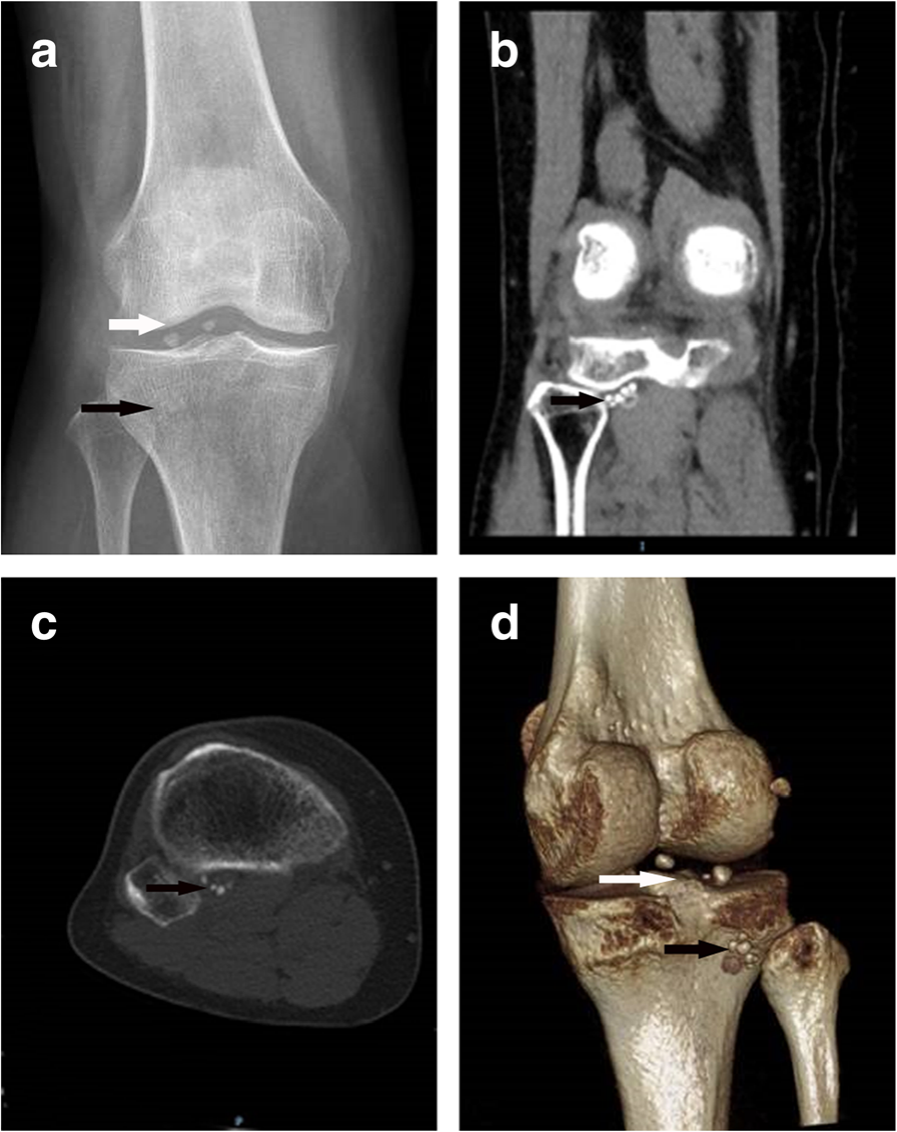
Images of loose bodies in the popliteus hiatus of the knee. Several ones (black arrows) were on the medial side of the fibular head, the other two (white arrows) were at the joint space. **a** A preoperative anteroposterior radiograph. **b** A coronal view of MRI. **c**, **d** Horizontal and three-dimensional rebuilding views of CT, respectively

**Fig. 2:**
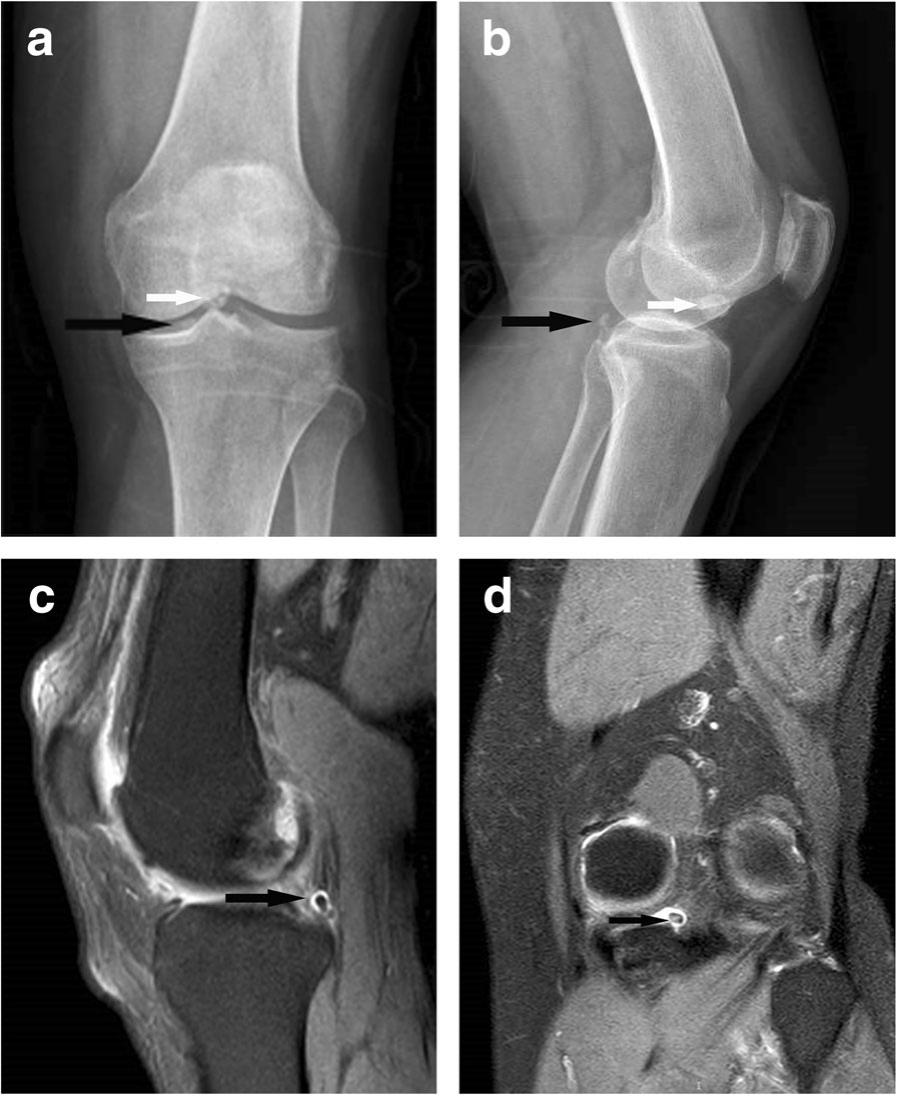
Images of loose bodies in the posteromedial compartment of the knee. One (black arrows) at the medial joint space, the other (white arrows) in the femoral intercondylar notch. **a**, **b** Preoperative anteroposterior and lateral views of the radiograph, respectively. **c**, **d** Sagittal and coronal views of MRI, respectively

Careful examination of the knee before the operation is essential before the operation. Comprehensive imaging examination is indispensable for the diagnosis of loose bodies (Figs. 1 and 2). Repeated X-ray films might provide confirmation of its mobility if loose bodies changed their locations. Three-dimensional computed tomography (CT) scanning is also needed to avoid missed diagnosis and show the spatial relationship of loose bodies. For loose bodies that are not radiopaque, magnetic resonance imaging (MRI) is vital and indispensable.

#### Routine arthroscopic examination

Firstly, a routine arthroscopic examination of the knee joint was performed using the standard anterolateral and anteromedial portals. These portals are made immediately adjacent to the lateral and medial border of the patellar tendon and 1 cm above the joint line when the knee is flexed 90°. Located loose bodies, especially the little ones, should be removed with straight hemostat or nucleus pulposus forceps for the fear that they move to another location. Meanwhile, concomitant meniscus injury is needed to be sutured or removed. Furthermore, hyperplastic synovium and osteochondral injury are needed to be tackled simultaneously.

#### Establishment of the accessory portals

**Fig. 3:**
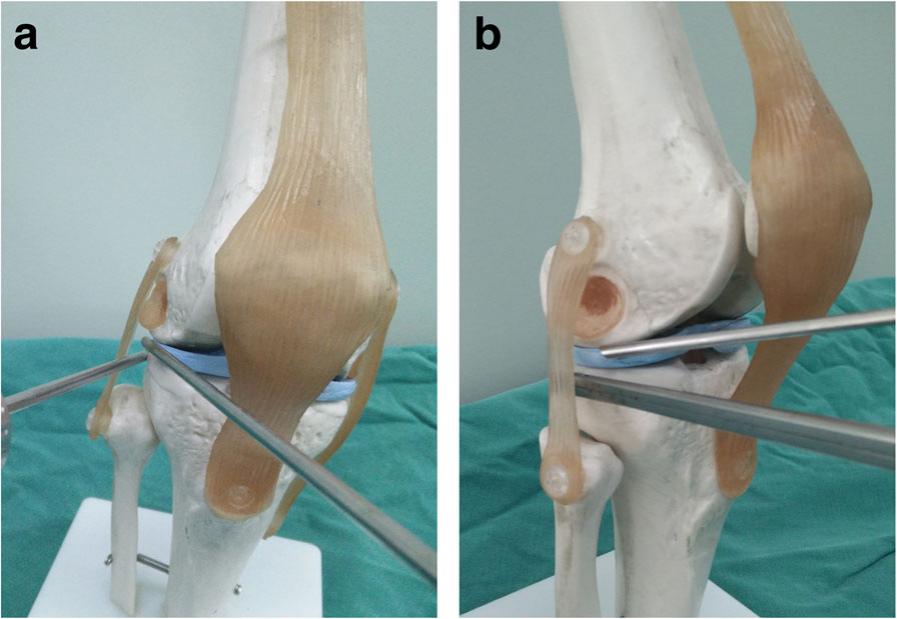
The model of an auxiliary extreme lateral approach combined with the conventional anterolateral approach in the knee. **a**, **b** Anterolateral and lateral views, respectively

**Fig. 4:**
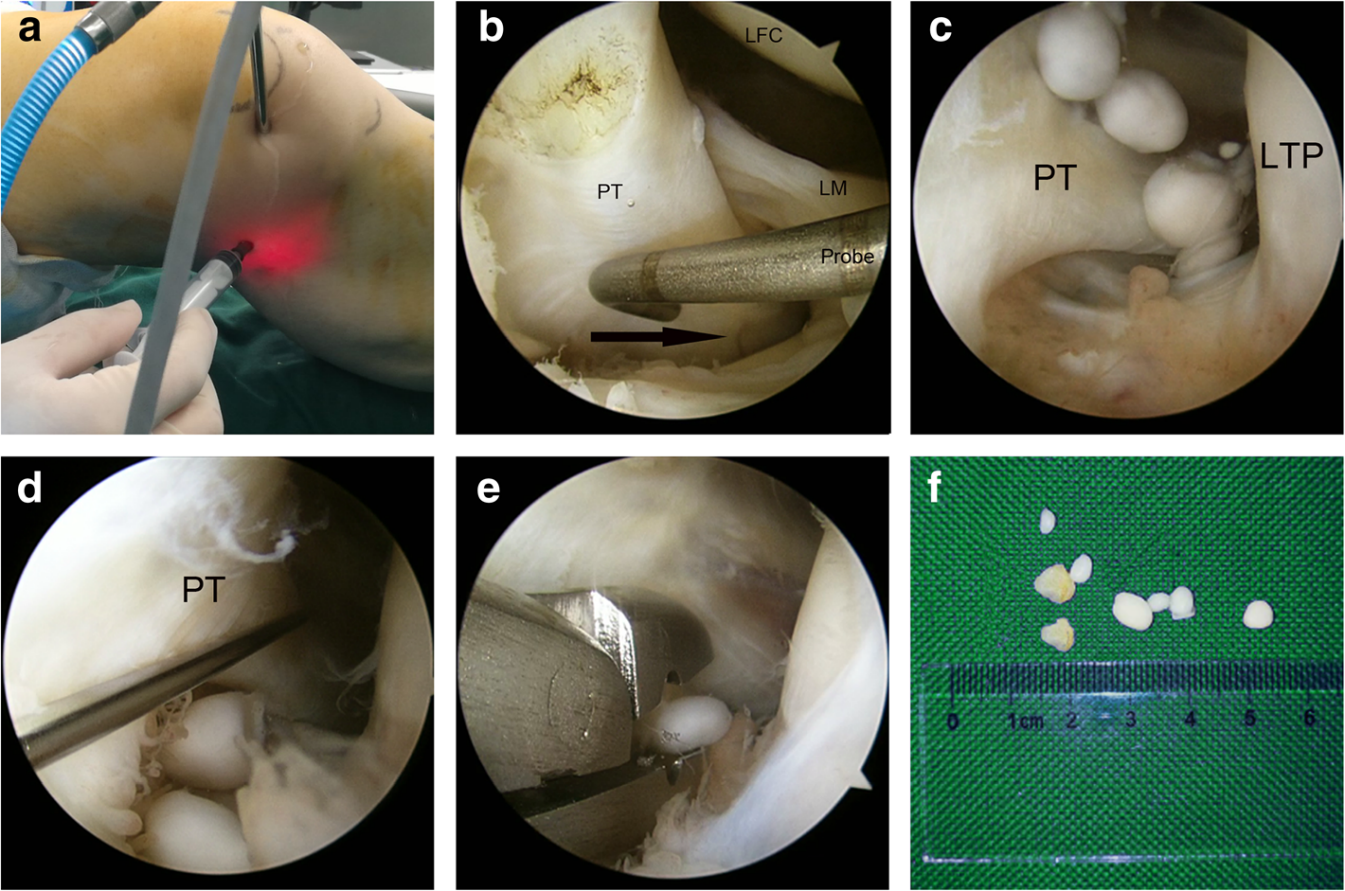
The establishment of an auxiliary extreme lateral approach. **a** The location of the auxiliary extreme lateral approach. **b**, **c** The arthroscopic finding of the popliteus tendon (PT) and popliteus hiatus as well as four loose bodies in the popliteus hiatus (PT, popliteus tendon; LTP, lateral tibial plateau). **d**, **e** The concealed loose bodies and their removal. **f** The eight removed loose bodies

**Fig. 5:**
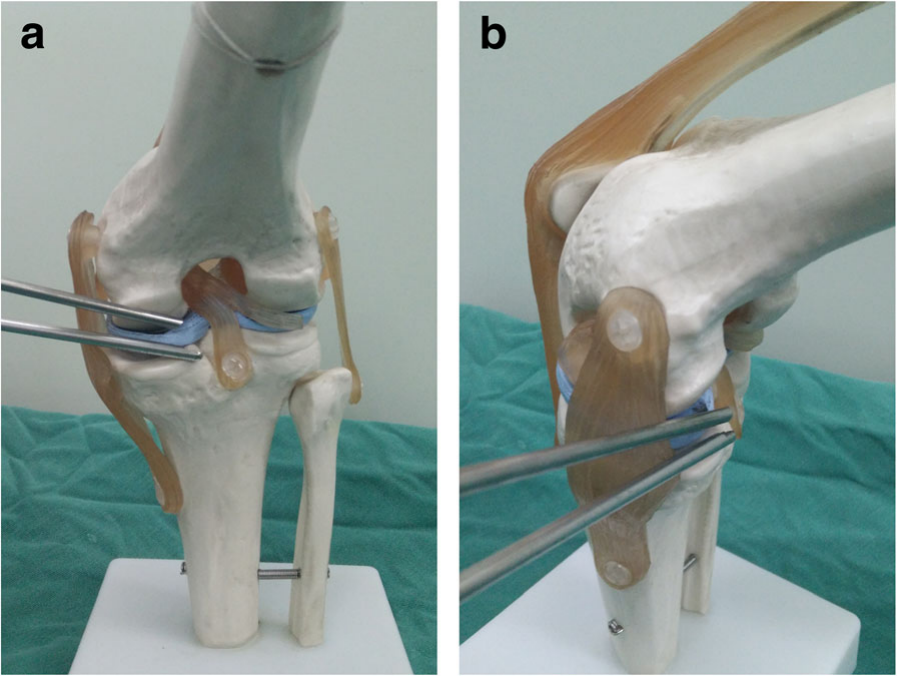
The model of a double-posteromedial portal in the knee. **a**, **b** Anterolateral and lateral views, respectively

**Fig. 6:**
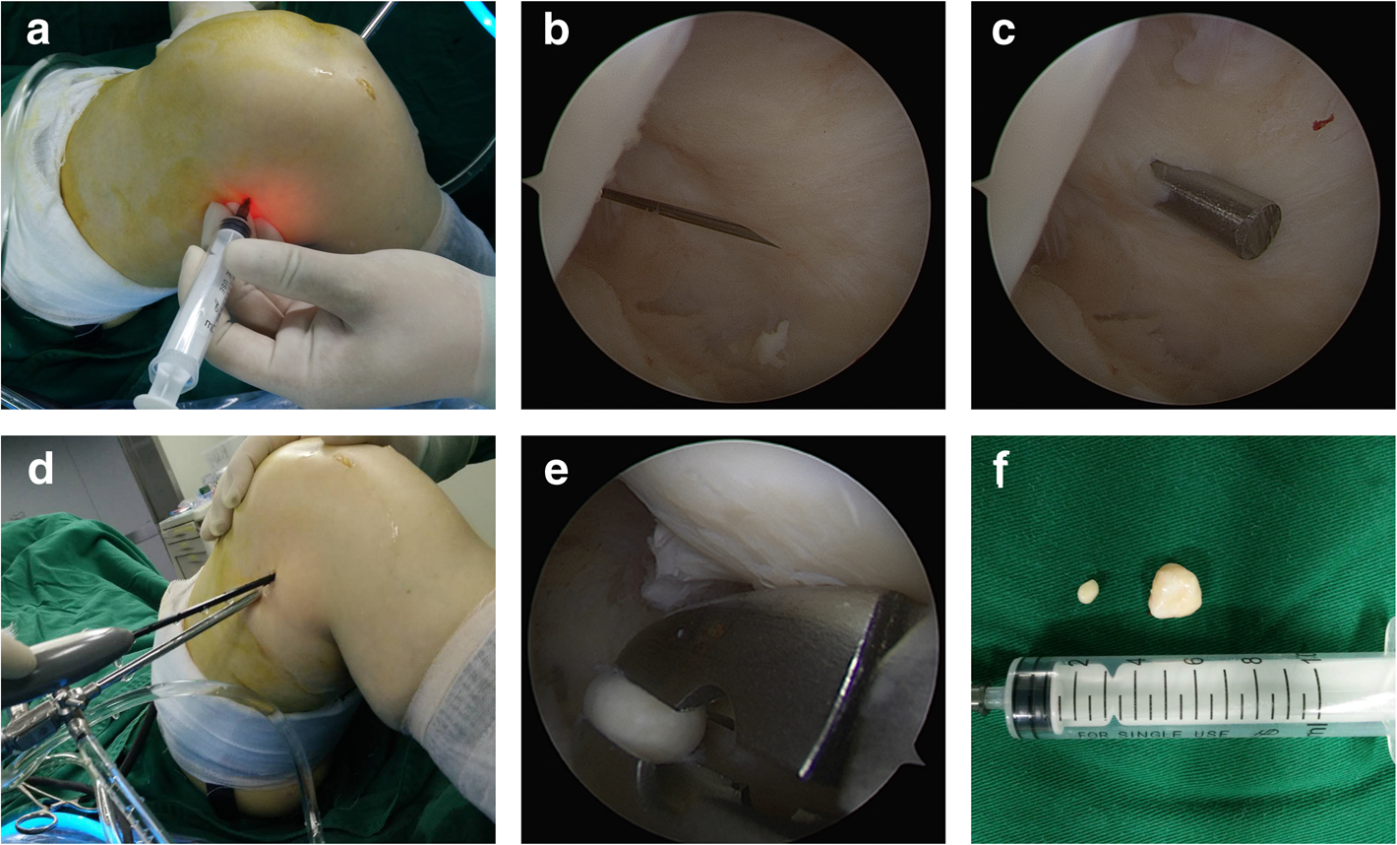
The establishment of a double-posteromedial portal. **a**, **b** The location of the lower posteromedial portal. **c** The location of the higher posteromedial portal (MFC, medial femoral condyle). **d** The location of a double-posteromedial portal. **e** The removal of a loose body through the lower posteromedial portal. **f** The two removed loose bodies

As is well known, it is often technically demanding to find and remove loose bodies located at several places, such as the popliteus hiatus and posterior compartment of the knee. Refractory loose bodies in our study were subdivided into three sections and were described as follows:

1. Popliteus hiatus: To remove loose bodies in popliteus hiatus, the knee is flexed to about 15 to 20° in a valgus position to keep the lateral soft tissue of the knee in a relaxed state (Fig. 3). Arthroscopy is introduced via the anterolateral portal and advanced to the lateral joint capsule. By using the light source of the arthroscopy for trans-illumination, the exact location for the popliteus hiatus is marked by a syringe needle. A minimal skin incision is made, establishing the auxiliary extreme lateral approach, which is about 1 cm above the joint line and 3 cm outside the anterolateral portal. This approach is used to explore the loose bodies in popliteus hiatus by the probe and thus remove those with nucleus pulposus forceps or straight forceps (Fig. 4). When necessary, the fingers squeeze the back of the small head of the fibula, and the remaining ones are squeezed out.
2. Posteromedial compartment: For loose bodies located in the posteromedial area, a double-posteromedial portal is required to be established (Fig. 5). The arthroscopy is moved to the anterolateral portal and advanced to the posteromedial compartment through the intercondylar notch between the posterior cruciate ligament and the lateral surface of the medial femoral condyle. As the arthroscopy advances to the posteromedial compartment, the knee is flexed to approximately 90°. By using the light source of the arthroscopy for trans-illumination, the exact location for the posteromedial portal is marked by a syringe needle. A minimal skin incision is made, establishing the first posteromedial portal, which is on the joint line and adjacent to the medial femoral epicondyle. The other portal is about 2 cm higher than the first one. The arthroscope is inserted from the higher posteromedial portal to provide a view of the posteromedial compartment of the knee. The lower posteromedial portal is used as an operating channel. If loose bodies are found, they can be removed by nucleus pulposus forceps or straight forceps through the lower posteromedial portal (Fig. 6).
3. Posterolateral compartment: As the previous literature described [3 13 14], with transnotch view from the anterolateral portal, the posteromedial portal is established firstly, and then, in the same way, the posterolateral portal is set up. Lastly, an aperture is made at the posterior septum, which is known as the posterior trans-septal portal technique.

### Statistical methods

SPSS 20 statistical software was used for statistical analysis. Descriptive statistics were used to report the data. Baseline patient characteristics were expressed as *n* for categorical variables and as the mean with the standard deviation for continuous variables. One-way analysis of variance (ANOVA) was used to compare demographics (age, height, and weight) among the three groups. A two-sided paired samples *t* test was used to compare the Lysholm score, Tegner score, and International Knee Documentation Committee (IKDC) score before and after surgery.

## Results

All the operations were successfully performed. Loose bodies are located in the suprapatellar bursa, intercondylar fossa, or joint space in 110 patients. Six patients were not followed up at 3 weeks after surgery. Seventy-six patients (24 males and 52 females, average age 54.9 ± 11.4 years) were followed up within 3 weeks. The mean height and weight were 161.7 ± 6.1 cm and 61.7 ± 6.2 kg, respectively (Table 1). There was no statistical difference among the three groups in demographics (age *p* > 0.05, height *p* > 0.05, weight *p* > 0.05). Patients were followed up at 3 weeks after surgery. Preoperative Lysholm, Tegner, and IKDC scores were 74.8 ± 4.2, 2.9 ± 0.87, and 56.4 ± 3.9, respectively. Postoperative 3 weeks of Lysholm, Tegner, and IKDC scores were 92.8 ± 3.7, 5 ± 0.96, 75.4 ± 4.7, respectively. There were significant differences between preoperative and postoperative scores (Lysholm *p* < 0.001, Tegner *p* < 0.001, IKDC *p* < 0.001), which indicated good prognosis (Table 2).

**Table 1:** Basic information

Demographics	Popliteus hiatus	Posteromedial	Posterolateral	Total
Patients (*n*)	20	40	16	76
Gender (M/F)	6/14	13/27	5/11	24/52
Age (years)*	57.9 ± 6.6	54.9 ± 13.9	53.3 ± 12.1	54.9 ± 11.4
Height (cm)*	160.2 ± 6.1	162.7 ± 6.5	161.1 ± 5	161.7 ± 6.1
Weight (kg)*	61.4 ± 7.3	62 ± 5	61.2 ± 8	61.7 ± 6.2

*M* male, *F* female

*The value is given as mean ± standard deviation

**Table 2:** Knee joint scores

Parameter (mean ± SD)	Preoperative	Postoperative	*p* value
Lysholm	74.8 ± 4.2	92.8 ± 3.7	< 0.001
Tegner	2.9 ± 0.87	5 ± 0.96	< 0.001
IKDC	56.4 ± 3.9	75.4 ± 4.7	< 0.001

*SD* standard deviation, *IKDC* International Knee Documentation Committee

## Discussion

We describe below the three locations of loose bodies and the technical aspects of the patients, who underwent surgery for symptomatic loose body removal in the popliteus hiatus and posterior compartment of the knee. Through our method, it is effective and safe to remove the loose bodies by the accessory portals under arthroscopy.

Loose bodies in several locations are difficult to remove. The popliteus hiatus is one of those locations. Multiple loose bodies are likely to locate at the popliteus hiatus due to its anatomical factors. Thus, it is very necessary to handle them at the same time. Few studies have introduced a specific surgical procedure to remove the loose bodies in the popliteus hiatus previously. Several strengths can be found in our method to remove loose bodies through the auxiliary extreme lateral approach. Firstly, a better arthroscopic visualization of loose bodies in the popliteus hiatus is possible, which was vital to handle under arthroscopy. Secondly, loose bodies in this location can be removed under direct arthroscopic view. Finally, it does not need to detach the lateral meniscus from the capsule to detect and remove the loose bodies in the popliteus hiatus, as described in a previous study [3]. However, when loose bodies are located more than 2–3 cm under the tibial plateau, they may be relatively hard to remove.

Loose bodies are commonly localized at the posterior compartment due to the gravity effect. However, it is often technically demanding to find and remove loose bodies located at the posterior compartment of the knee joint arthroscopically. Loose bodies in most of our patients are found in the posteromedial compartment of the knee. Boytim et al. [14] evaluated the posteromedial visualization of the knee via an anterolateral portal. Ahn et al. [15] as well as Pengatteeri et al. [13] used the trans-septal portal and posterior–posterior triangulation techniques to remove the loose bodies in the posteromedial area, respectively. However, there are still some limitations to handle loose bodies using previous methods. Thus, in the current study, we choose a double-posteromedial portal to remove the loose bodies in the posteromedial compartment of the knee. There exist several advantages to our method. First of all, loose bodies can be visualized and removed in this way. Furthermore, the neurovascular structure is mostly located in the posterolateral compartment of the knee, while all of our operative procedures are in the posteromedial compartment. It is a safer method compared with the trans-septal portal posterior–posterior triangulation techniques. Finally, our method does little harm to the posterior septum, which contained several neurovascular structures and mechanoreceptors. The preservation of the superior part of the posterior septum can maintain a greater number of blood vessels and mechanoreceptors to improve proprioception [16–18].

As for the loose bodies in the posterolateral compartment, the trans-septal portal posterior–posterior triangulation techniques might be a better way, just as described in the previous studies [3, 15, 19]. There are some keys as follows: first, it is safe to establish posterior and trans-septal arthroscopic portals in the position of 90° of knee flexion [11]. Pace and Wahl [20] thought that there was an adequate safe zone between the popliteal neurovascular structures and the posterior capsule with the knee flexed to 90°. Besides, if debridement of the posterior septum is indispensable, it should be done at the inferior aspect so that a greater number of blood vessels and mechanoreceptors can be preserved [16].

Preoperative imaging examination is essential for preoperative planning. X-ray is one of the diagnostic criteria for loose bodies of the knee. Sometimes, repeated X-ray may be necessary to determine the position of loose bodies, as well as to distinguish loose bodies from the calcification of ligament. However, some authors [3, 6] thought that the absence of loose bodies on X-ray did not rule out its presence in the knee. Thus, some loose bodies might be easily missed only through lateral and anteroposterior X-ray. Currently, CT and MRI are the procedures of choice in evaluating the loose bodies. CT may be useful to find tiny loose bodies and show the spatial relationship of them, while MRI can be used to discover non-radiopaque loose bodies. Besides, MRI may find some loose bodies that are difficult to examine arthroscopically. Gold et al. [21] concluded that some presumed false-positive MRI readings might reflect an incomplete arthroscopic examination rather than a failure of radiologic diagnosis. Also, MRI is of great value in the diagnosis of knee joint articular cartilage, meniscus, ligaments, bone, and another soft tissue injury, which can help to formulate a more comprehensive preoperative plan. It is important to find loose bodies located at the popliteus hiatus and posterior compartment of the knee with the combination of the three imaging methods.

Knee joint scores were significantly improved at postoperation than preoperative data with increased Lysholm, Tegner, and IKDC scores. We have experienced good clinical results after the removal of difficult loose bodies in the popliteal hiatus and posterior compartment. Furthermore, it is simple and convenient to achieve loose body removal in the difficult locations with the accessory portal techniques.

Our current study has some limitations. First, this study was a retrospective research. Secondly, our sample size was relatively small, and the follow-up was relatively short. In the future, a prospective randomized controlled trial with a larger sample size and a longer follow-up will be performed to evaluate the effectiveness and conciseness of the accessory portal techniques.

## Conclusions

Although it is quite often technically challenging, loose bodies could be removed even in the most difficult locations with the help of accessory portals. Also, planning by repeated X-rays and preoperative MRI can be a guide to find loose bodies in the knee.

## Abbreviations

ANOVA: One-way analysis of variance
CT: Computed tomography
IKDC: International Knee Documentation Committee
MRI: Magnetic resonance imaging
OA: Osteoarthritis
